# Angle-of-approach and reversal-movement effects in lateral manual interception

**DOI:** 10.3389/fpsyg.2024.1433803

**Published:** 2024-11-07

**Authors:** Simon Ledouit, Danial Borooghani, Remy Casanova, Nicolas Benguigui, Frank T. J. M. Zaal, Reinoud J. Bootsma

**Affiliations:** ^1^Institut des Sciences du Mouvement, Aix-Marseille Université, CNRS, Marseille, France; ^2^GREYC, Normandie Univ, UNICAEN, CNRS, Caen, France; ^3^Department of Human Movement Sciences, University Medical Center Groningen, Groningen, Netherlands

**Keywords:** perceptuomotor, control, interception, timing, information, visual guidance

## Abstract

The present study sought to replicate two non-intuitive effects reported in the literature on lateral manual interception of uniformly moving targets, the angle-of-approach (AoA) effect and the reversal-movement (RM) effect. Both entail an influence of the target trajectory’s incidence angle on the observed interceptive hand movements along the interception axis; they differ in the interception location considered. The AoA effect concerns all trajectory conditions requiring hand movement to allow successful interception, while the RM effect concerns the particular condition where the target will in fact arrive at the hand’s initial position and no hand movement is therefore required but nevertheless regularly produced. Whereas the AoA effect has been systematically replicated, the RM effect has not. To determine whether the RM effect is in fact a reproducible phenomenon, we deployed a procedure enhancing the uncertainty about the target’s future arrival locations with respect to the hand’s initial position and included low-to-high target motion speeds. Results demonstrated the presence of both the AoA effect and the RM effect. The AoA effect was observed for all relevant interception locations, with the effect being stronger for the farther interception locations and the lower target speeds. The RM effect, with the hand first moving away from its initial position, in the direction of the target, before reversing direction, was observed in a higher proportion of trials for target trajectories with larger incidence angles and lower speeds. Earlier initiation gave rise to reversal movements of larger amplitude. Both effects point to visual guidance of hand movement partially based in reliance on information with respect to current lateral ball position. We conclude that the information used in lateral manual interception is of an intermediate order, which can be conceived as resulting from a partial combination of target position and velocity information or information in the form of a fractional order derivative.

## 1 Introduction

At first sight the act of reaching out to catch an approaching ball appears to be quite simple. There is to date, however, still no consensus on the perceptuomotor processes underlying the performance of such an elementary action. One of the research paradigms developed to facilitate analysis is that of lateral manual interception, in which the hand is constrained to move along a predefined interception axis. Within this paradigm experimental control of the trajectories of the balls to be caught allows studying the kinematic patterns of the interception movements, with the end-goal of each movement (i.e., when the hand should be where) being experimentally defined. In order to avoid the uncontrolled and relatively large inherent variability in the spatiotemporal characteristics of ball trajectories resulting from the use of launching devices, Peper et al. (1994) had participants catch balls, suspended with fishing line from a high ceiling, swinging down from an initial holding position. Combining (laterally) different suspension points and initial positions, this setup allowed them to confront participants not only with balls arriving at different (experimentally controlled) locations along the interception axis, but also with balls arriving at the same interception location after the same flight duration while coming from different starting positions.

This first systematic study of lateral manual interception brought out an influential result: even though participants caught the ball on all trials, the kinematics of the interception movements varied over ball trajectories converging onto the same interception location. This result, labeled the *angle-of-approach effect* by Peper et al. (1994), clearly contradicts a predictive type of control based on *a priori* perceptual estimates of when the ball will be where, as both the when and the where were invariant over the ball trajectories converging onto the same interception location. To account for the pattern of results obtained, Peper et al. (1994) suggested that control was prospective, rather than predictive, and proposed a model in which hand movement is continuously regulated on the basis of information with respect to currently required hand velocity. The latter was defined as the (time evolving) ratio of the distance between the current lateral positions of the ball and hand to the time remaining until the ball arrived at the interception axis: interception is ensured when the lateral distance between ball and hand reaches zero at the moment the ball crosses the interception axis. Peper et al. (1994)’s finding that spatial ball trajectory characteristics affect the kinematics of lateral manual interception movements has been systematically replicated in studies using pendular (Dessing et al., 2005, 2009a,b; Jacobs and Michaels, 2006; Michaels et al., 2006) or rectilinear (Montagne et al., 1999; Arzamarski et al., 2007; Ledouit et al., 2013) ball trajectories (also see Bootsma et al., 2016, for a similar finding in lateral locomotor interception). This body of research thus provides a firm empirical basis for the claim that lateral interception is based on prospective rather than on predictive control. However, what exactly the information is that is used to guide the hand to the future interception location has yet to be established.

**Figure 1:**
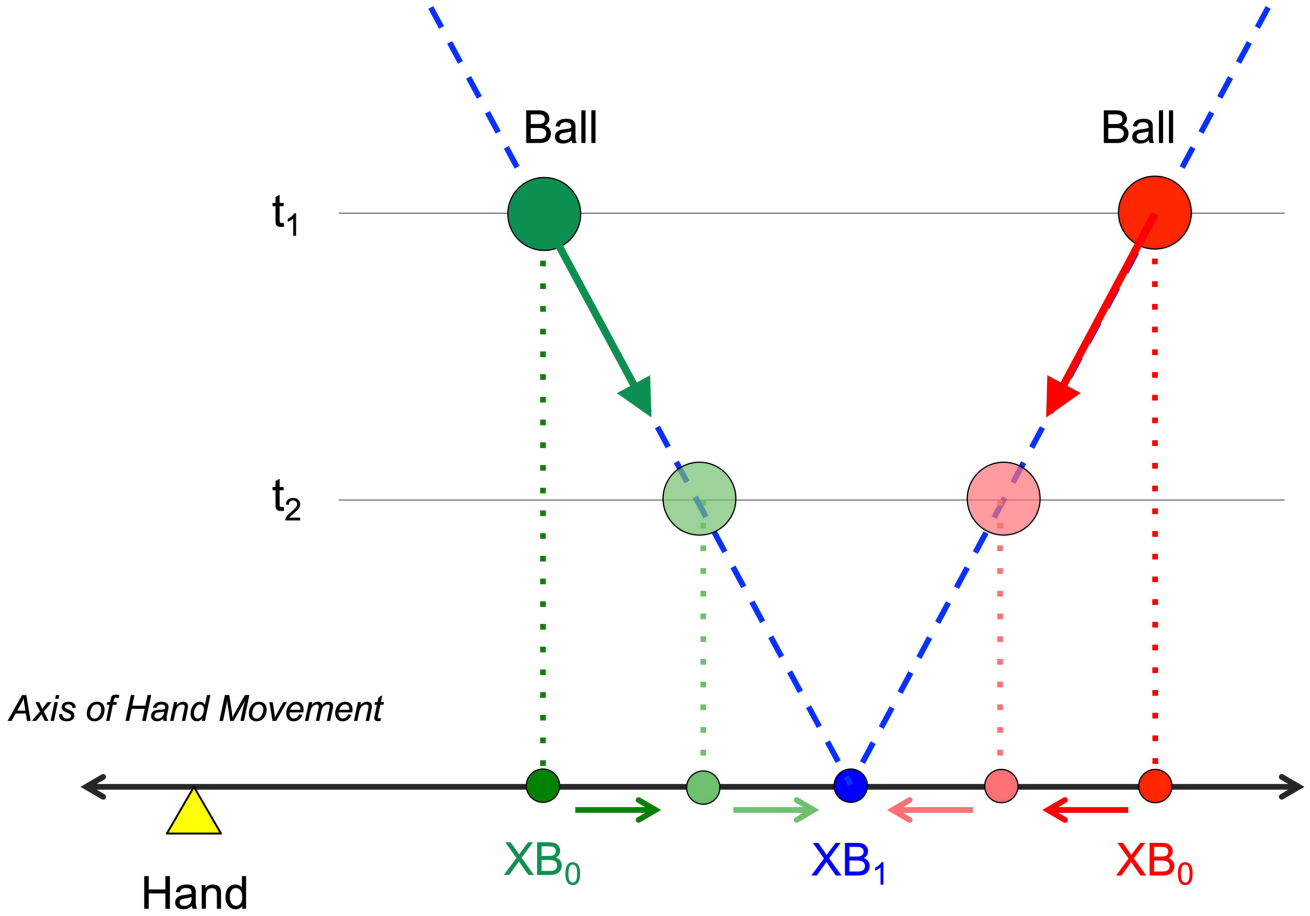
Definition of variables *XB*_0_ and *XB*_1_. *XB*_0_ is the current lateral position of the ball projected orthogonally on the interception axis (along which the hand can move). *XB*_1_ is the future lateral position of the ball on the interception axis if current heading is maintained. Balls (green and red circles) moving along rectilinear trajectories (dashed blue lines) with constant velocity (fat green and red arrows) will cross the interception axis at position *XB*_1_ (blue circle). While, for a given ball trajectory, *XB*_1_ is invariant over time, *XB*_0_ evolves over time, moving either inward (red) or outward (green) toward the future ball arrival position *XB*_1_, as a function of the ball-trajectory incidence angle with the interception axis.

In the framework of lateral manual interception, the relevant (physical) properties of the Environment-Agent System (EAS, see Bootsma, 1998) generally considered are the ball’s current lateral position (*XB*_0_), the position on the interception axis toward which it is currently heading (*XB*_1_, see Figure 1 for definitions of *XB* variables) and the current time it will take before it reaches the interception axis (i.e., first-order time to contact *TC*_1_). Analyses of the optical information specifying these properties have been performed for (uniform) rectilinear ball motion in the transverse plane located at the observer’s eye-height (e.g., Lee, 1976; Todd, 1981; Bootsma, 1991; Bootsma and Peper, 1992; Bootsma and Oudejans, 1993; Regan and Kaushal, 1994; Bootsma and Craig, 2002; Gray and Regan, 2004; Gray and Sieffert, 2005; Jacobs and Michaels, 2006; Michaels et al., 2006). These analyses have demonstrated that the above listed physical quantities are indeed specified by the states of a ball’s azimuthal bearing angle *θ* and its optical size *φ*. For instance, (under the assumption of small angles) for a ball of a given constant size the ratio of *θ* to *φ* (i.e., *θ*/*φ*) is specific to the ball’s current lateral distance (*XB*_0_) and the ratio of *dθ/dt* to *dφ/dt* (i.e., (*dθ/dt)/(dφ/dt*)) is specific to the lateral position at which it will pass the interception axis (*XB*_1_). Because from a bird’s-eye view pendular trajectories produce linear motion in the participant’s transverse plane, it was generally assumed that the above-mentioned informational variables indeed allow participants to access physical variables such as *XB*_0_, *XB*_1_, and *TC*_1_. Michaels et al. (2006) demonstrated, however, that pendular trajectories differ from rectilinear trajectories in terms of the evolution of the states of the optic variables that might be used. For instance, rather than being invariant over time as it is for a rectilinear ball trajectory, (*dθ/dt)/(dφ/dt*) varies during approach of a ball following a pendular trajectory. The relation between potential optical information sources [e.g., *θ/φ* and (*dθ/dt)/(dφ/dt*)] and the physical variables they are presumed to specify (e.g., *XB*_0_ and *XB*_1_) is thus distorted for balls following pendular trajectories, making straightforward interpretations of the results observed considerably more difficult. Because the relations remain direct (i.e., univocal) for rectilinear ball trajectories, in the present contribution we focus on the lateral manual interception of balls following rectilinear trajectories.

Inspired by Peper et al. (1994)’s findings and interpretation, Montagne et al. (1999) set out to test two predictions of the required-velocity model using a lateral manual interception task with balls approaching in the transverse plane along rectilinear trajectories. The first prediction was directly derived from Peper et al. (1994): if the hand was controlled on the basis of information about the ball’s future arrival position *XB*_1_ (in this case univocally specified by (*dθ/dt)/(dφ/dt*)), then trajectories converging onto the same lateral position on the interception axis should give rise to identical patterns of hand movement. If, however, the hand was controlled on the basis of information about the ball’s current lateral position *XB*_0_ (now univocally specified by *θ/φ*), then rectilinear trajectories converging onto the same lateral position on the interception axis should give rise to distinct, trajectory-specific patterns of hand movement. The second prediction was also based on divergent expectations stemming from the use of information about *XB*_0_ or *XB*_1_, but called upon an experimental condition hitherto not explored: balls arriving at the initial hand position should not give rise to hand movement if the hand was controlled on the basis of information about the ball’s future arrival position *XB*_1_. However, if the hand was controlled on the basis of information about the ball’s current lateral position *XB*_0_, then for balls approaching the interception axis via oblique trajectories one should expect the hand to first move away, in the direction of the current lateral ball position, before returning to the initial hand position to catch the ball.

Montagne et al. (1999) reported two major results in favor of *XB*_0_-based control. First, balls following different trajectories (i.e., starting from different ball departure positions) but arriving after the same duration of motion at the same location on the interception axis (i.e., identical ball arrival positions) were demonstrated to give rise to ball trajectory-dependent kinematic interception patterns (i.e., different hand position profiles), the phenomenon referred to as the *angle-of-approach effect* (*cf.* Peper et al., 1994). Second, while hand movement was clearly not required to intercept balls arriving at the initial hand position, in 50% of the trials movement of the hand was nevertheless observed under such conditions, with the hand moving away from its initial position to subsequently return there to catch the ball. These *reversal movements* moreover did not appear to be random in their initial direction: balls coming from a starting position on the left tended to evoke left–right reversal movements and balls coming from a starting position to the right tended to evoke right–left reversal movements. Neither of these results is consistent with an organization of movement based on predictive control nor on prospective control based on information about *XB*_1_.

In an attempt to replicate Montagne et al. (1999)’s results, Arzamarski et al. (2007) studied lateral manual interception of balls rolling across a solid surface (and thus following rectilinear trajectories in the transverse plane). While their findings replicated the angle-of-approach effect, they did not observe the number, nor the pattern of reversal movements reported by Montagne et al. (1999). This led them to suggest that participants relied on *XB*_1_-specific information, proposing that the angle-of-approach effect in fact resulted from a perceptual bias in the (presumably ongoing) extrapolation of the ball trajectory to the future arrival position on the interception axis. Studying lateral manual interception of simulated balls following rectilinear trajectories along a screen oriented in the participants’ fronto-parallel plane, Ledouit et al. (2013) demonstrated that the angle-of-approach effect generalized to this new experimental situation. In a series of follow-up experiments Ledouit et al. (2013) subsequently demonstrated that this effect could not be explained by Arzamarski et al. (2007)’s perceptual bias hypothesis, as controlling for this extrapolation bias did not lead to the disappearance of the angle-of-approach effect. Yet, while the angle-of-approach effect has thus systematically been observed when participants intercept balls moving along different rectilinear trajectories converging onto the same interception location, the number and pattern of reversal movements reported by Montagne et al. (1999) was observed neither by Arzamarski et al. (2007) nor by Ledouit et al. (2013).

How then should we consider the pattern of reversal movements reported by Montagne et al. (1999)? The fact that, contrary to the angle-of-approach effect, reversal movements were not observed in other studies and were not even systematically observed by Montagne et al. (1999) led Arzamarski et al. (2007) to question their very existence as a reproducible experimental observation. However, before concluding that the reversal movements reported by Montagne et al. (1999) simply cannot be reproduced, their experimental procedure merits to be more thoroughly scrutinized. In doing so, we also include what we consider to be pertinent elements that were not fully reported in the original publication.

In Montagne et al. (1999)’s study the balls to be caught were attached to a pole extending above and in front of a motorized cart that moved at constant speed along a straight track. Different angles of approach to a particular position on the participants’ interception axis were obtained by rotating the whole track around a vertical axis located just below the interception axis. With balls thus always arriving at the same physical interception point, different interception conditions were obtained by positioning both the participant’s hand and feet at different locations at the start of each trial. To this end, three different initial hand positions were combined with two different foot positions. It is important to realize that throughout this experiment participants wore opaque liquid crystal spectacles that were only switched to their transparent state when the ball was approaching the interception axis; they switched back to their opaque state as soon as the ball arrived there. When participants were asked to move their hand and feet to the subsequent trial’s initial conditions, they thus did so without vision of the environment: the experimenter in fact physically guided them to the position to be adopted. Due to “black light” illumination during ball approach, participants could moreover only see the white ball and the white glove on their hand. We suggest that this elaborate procedure prevented participants from knowing where the ball was going to arrive, as they did not know themselves where they were in the environment.

In both Arzamarski et al. (2007) and Ledouit et al. (2013) studies participants sat or stood in the same place for the duration of the experiment. In Arzamarski et al. (2007) study balls could arrive at one of three arrival positions. These three arrival positions were moreover marked on the table, as they corresponded to the three initial hand positions that participants could be asked to adopt at the start of a given trial. In Ledouit et al. (2013) study participants always started from the same initial position while balls could arrive at five different arrival positions, including the initial hand position. Neither of these studies thus created the same kind of uncertainty with respect to where the ball could arrive as did Montagne et al. (1999)’s study.

The goal of the present study was therefore to create the conditions that might lead to the emergence of reversal movements like the ones reported by Montagne et al. (1999). Rather than replicating Montagne et al. (1999)’s elaborate procedure, we modified the virtual interception task of Ledouit et al. (2013) in the following ways. First, in order to enhance uncertainty with respect to the initial hand position, at the start of every trial the participant was required to position the hand-held stylus in a small rectangle appearing in randomly selected positions along the interception axis. When this (random) position was correctly attained, the stylus was guided to the trial’s initial position by slowly moving the rectangle. Thus, while in the end the hand was always in the same position at the onset of ball motion (identical to the one used in Ledouit et al., 2013’s, study), the initial positioning procedure was expected to induce at least some degree of uncertainty. Second, we used seven rather than five different arrival positions: while in Ledouit et al. (2013)’s study balls arrived at the initial hand position in 1/5th or 20% of the trials, this probability was now reduced to 1/7th or 14% of the trials. Moreover, the closest arrival positions on either side of the initial hand position were now also located closer to the initial hand position than in the Ledouit et al. (2013) study. Finally, we used a range of (orthogonal) ball speeds chosen so that interception became quite difficult at the highest ball speed (or, put differently, at the shortest ball flight time). As ball speed randomly varied over trials, inclusion of such a high ball speed (i.e., short ball flight time) condition, requiring early initiation to allow successful interception, was intended to evoke relatively early onsets of interceptive movements under all ball speed condition as a result of operation of the range effect (Poulton, 1975). Since we expect current lateral distance *XB*_0_ to play a role in the control of lateral manual interception movements, relative early movement initiation would bring this effect out most clearly, as the lateral distance between ball departure and ball arrival positions is then largest (compare t_1_ and t_2_ in Figure 1).

With these distinctive procedural adaptations to Ledouit et al. (2013)’s study, we aimed to not only evoke angle-of-approach effects but also reversal movements as reported by Montagne et al. (1999).

## 2 Methods

### 2.1 Participants

Ten right-handed students and junior staff members from Aix Marseille University, 5 men and 5 women (22.6 ± 3.2 years old, M ± SD), voluntarily took part in the experiment. All participants were free from known motor impairments and reported normal or corrected-to-normal vision. Before the start of the first experimental session the participants were informed about the aim and procedure of the experiment. All participants provided written informed consent before participating in the study. The study was approved by the local institutional review board (*Comité Ethique de l’Institut des Sciences du Mouvement d’Aix-Marseille Université*) and conducted according to University regulations and the Declaration of Helsinki. For the record we note that the (thus far unpublished) data were collected by author SL in 2014, in the framework of his doctoral thesis research program. Author DB fully reanalyzed the original dataset in preparation of the present contribution.

### 2.2 Task and procedure

**Figure 2:**
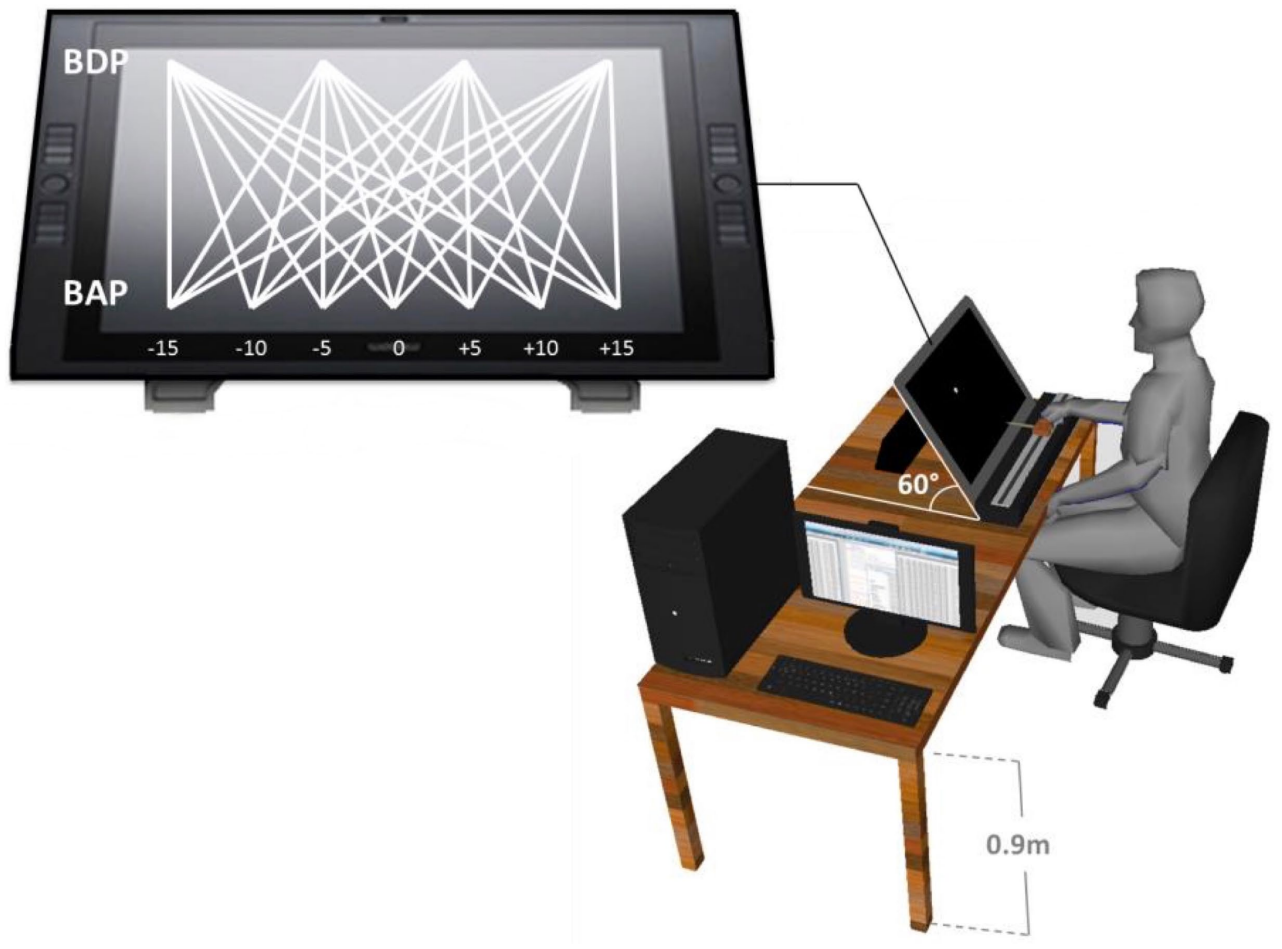
Representation of the experimental set-up. Participants moved the stylus along the (horizontal) interception axis to intercept virtual balls moving from one of four Ball Departure Positions (BDP) to one of seven Ball Arrival Positions (BAP).

The experiment took place in a darkened room without windows. The participant sat in a chair in front of an interactive Cintiq 21UX Wacom^®^ tablet (screen size 43.2 × 32.4 cm, 1,600 × 1,200 pixel resolution) positioned at a height of 0.90 m and oriented at a 60° angle, providing a plane of motion perpendicular to the participant’s line of sight (*cf.* Ledouit et al., 2013). The task was to intercept simulated balls, moving downward (top-to-bottom) across the tablet’s screen, by laterally displacing the tablet’s stylus along the bottom of the screen. The stylus was rigidly attached to a hand-held knob that could slide over a horizontal 50-cm long rail fixed to the table in front of the participant. Participants had full vision of both their hand on the knob and the stylus with its point touching the screen (see Figure 2).

A trial began with the appearance of a 1.5-cm wide by 0.8-cm high red rectangle at a random position, between −10 and + 10 cm from the center, on the interception axis near the bottom of the screen. When the participant positioned the stylus within this rectangle it became green and slowly (5 cm/s) moved to the trial’s starting position. Participants were required to maintain the stylus within the rectangle until it came to a stop and stay at this position after the rectangle disappeared, until 1 s later a ball appeared at the top of the screen. Even though the starting position was in fact always the same (at the center of the screen), this procedure of leading the participant on every single trial over a variable distance to a designated starting position was intended to suggest that starting position could vary. The X-Y origin of the screen was defined with respect to the starting position, X increasing negatively to the left and positively to the right of the starting position and Y increasing positively to the top of the screen. The interception trial started when a ball, represented by a 1.6-cm diameter white circle against a black background, appeared at one of the four possible departure positions (Y = +32 cm; X = −15, −5, +5, or + 15 cm) and moved at constant velocity across the screen toward one of seven possible arrival positions along the interception axis (Y = 0 cm; X = −15, −10, −5, 0, +5, +10, or + 15 cm). Combining the four Ball Departure Positions (BDP) and the seven Ball Arrival Positions (BAP) gave rise to 28 different rectilinear ball trajectories. Balls could move at constant orthogonal (Y) speeds of 16, 20, 26.67, or 40 cm/s, for Ball Flight Times (BFT) of 2.0, 1.6, 1.2, or 0.8 s until crossing the interception axis. For reasons of readability, we will hereafter generally refer to the lowest-to-highest orthogonal ball speeds as BS1, BS2, BS3 and BS4, respectively. Participants performed 3 blocks of 112 trials (28 trajectories × 4 ball speeds), with the order of conditions randomized over trials within each block. Feedback with respect to interception (yes/no) was automatically provided at the end of each trial, with successful interception requiring that the distance between stylus and ball center was less than 0.8 cm (equivalent to the ball radius). During the experiment, ball and stylus positions were sampled at a frequency of 100 Hz and stored on disk for each individual trial.

### 2.3 Data analysis

Due to recording problems, 60 of the 3,360 experimental trials (i.e., 1.8%) had to be excluded from the dataset, leaving a total of 3,300 trials for analysis. The stylus position time-series were filtered using a second-order Butterworth filter with a cut-off frequency of 5 Hz. Interception performance was assessed using success rate and absolute error, with the latter defined as the unsigned distance between ball and stylus positions at the moment the ball crossed the interception axis. Absolute error was statistically analyzed using a repeated-measures Analysis of Variance (ANOVA) with factors of Ball Speed (BS, 4 levels), Ball Departure Position (BDP, 4 levels), and Ball Arrival Position (BAP, 7 levels). General trends in movement kinematics were captured in ensemble averages of the time series of position of stylus displacement. In order to statistically test differences in movement kinematics, we analyzed the moment of movement initiation (MoI), operationally defined as the first moment that hand velocity exceeded 1 cm/s when searching back in time from the moment peak velocity was attained, as well the magnitude of the peak velocity (PV) reached. These latter variables were statistically analyzed using repeated-measures Analyses of Variance (ANOVA) with factors of Ball Speed (BS, 4 levels), Ball Departure Position (BDP, 4 levels), and Ball Arrival Position (BAP, 6 levels with BAP = 0 excluded). Greenhouse-Geiser corrections were applied whenever the assumption of sphericity was violated (Mauchly’s W-test). When appropriate, significant (*α* = 0.05) main effects and interactions were further analyzed using Tukey post-hoc tests. ANOVAs were performed on the participant means over trials of identical BS × BDP × BAP conditions.

For the 4 BDP × 4 BS conditions arriving at BAP = 0 cm (i.e., at the initial stylus position) we determined the number, moment of initiation and amplitude of reversal movements. A reversal movement was defined as a first movement in one direction, with a minimal displacement of 0.8 cm (i.e., ball radius) and a velocity exceeding 1 cm/s, followed by change in direction reaching at least a 1-cm/s velocity. The amplitude of a reversal movement was defined as the peak position of the first identified movement and its moment of initiation as the first time velocity exceed 1 cm/s toward this peak position.

## 3 Results

### 3.1 Performance

Overall, participants intercepted 80.0% of the balls. As was to be expected, interception rates varied with orthogonal ball speed (and, therefore, with ball flight duration); interception rates were 94.2, 91.9, 80.1, and 53.9%, for the slowest to fastest ball speeds, respectively. This decrease in interception performance with increasing ball speed was also observed at the level of Absolute Error, for averages of 0.34, 0.40, 0.53, and 0.99 cm, respectively. The ANOVA on Absolute Error revealed main effects of BS [*F*(3, 27) = 42.81, *p* < 0.001, η^2^*_p_* = 0.83], BDP [*F*(3, 27) = 3.57, *p* = 0.023, η^2^*_p_* = 0.29], and BAP [*F*(6, 54) = 34.57, *p* < 0.001, η^2^*_p_* = 0.79], as well as significant two-way interaction effects of BS × BDP, *F*(9, 81) = 4.00, *p* < 0.001, η^2^*_p_* = 0.31, BS × BAP, *F*(18, 162) = 12.61, *p* < 0.001, η^2^*_p_* = 0.58, and of BDP × BAP, *F*(18, 162) = 11.63, *p* < 0.001, η^2^*_p_* = 0.56. *Post hoc* analysis of the overarching 3-way interaction [*F*(54, 486) = 1.70, *p* = 0.002, η^2^*_p_* = 0.16] indicated that Absolute Error was essentially larger for balls traveling at highest speed (BS4) or, more rarely, at the slightly slower speed (BS3) to the furthest BAPs (−15 and + 15 cm) while coming from opposite BDPs.

### 3.2 Movement kinematics part 1: trajectory effects when interception requires movement

Because balls arriving at BAP = 0 cm did not require participants to move (as the ball arrival position corresponded to the initial stylus position), we will treat this particular BAP condition in a separate section. Here we focus on the six BAP conditions that did require participants to move in order to intercept the ball (i.e., BAP = −15, −10, −5, +5, +10, and + 15 cm).

**Figure 3:**
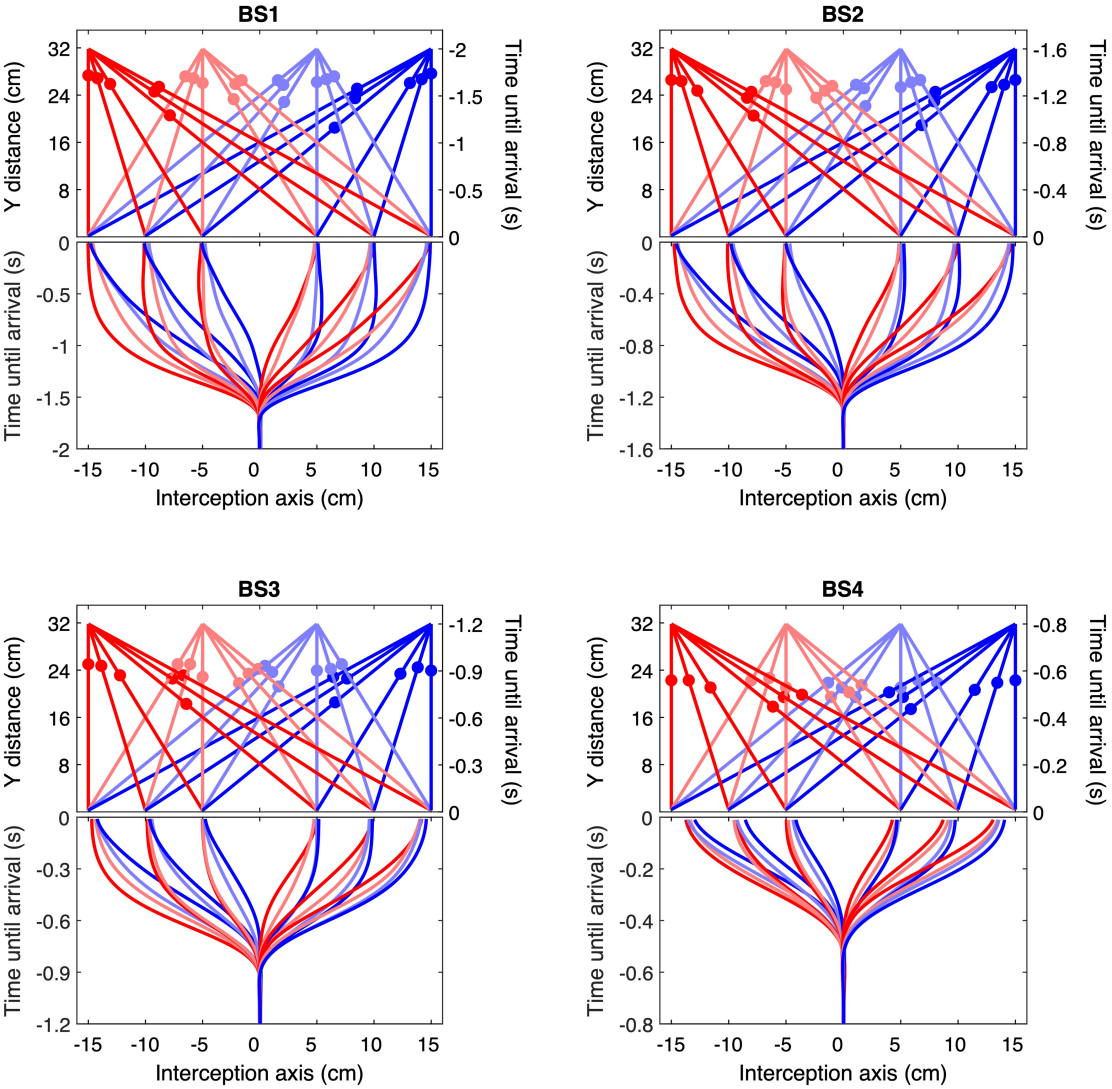
Ball trajectories and corresponding ensemble average interception movements for each of the four Ball Speed conditions (with ball flight times of 2.0, 1.6, 1.2, and 0.8 s for BS1–BS4, respectively). The upper part of each panel shows the 24 rectilinear trajectories arriving at positions −15, −10, −5, +5, +10, and + 15 cm, with filled circles indicating ball position at the mean moment of initiation of the interception movement. Each color corresponds to a common ball departure position. The lower part of each panel presents the ensemble averages of stylus position as a function of time until ball arrival, using the same color codes as in the upper part. Please note the differences in (vertical) time scale in the four panels.

Ensemble averages of stylus position over time for the six BAPs requiring stylus movement are presented in Figure 3, for each of the four ball speed conditions separately. Visual inspection of Figure 3 indicated several noteworthy results. These observations were corroborated by post-hoc analyses of the overarching triple interactions observed in repeated-measures three-way ANOVAs (factors BS, BDP, and BAP) on the pertinent variables (see Table 1).

**Table 1:** ANOVA results for the moment of movement initiation (MoI) and magnitude of peak velocity (PV).

			MoI			|PV|	
Source	DoF	*F*	*p*	η^2^*_p_*	*F*	*p*	η^2^*_p_*
BS	3/27	194.9	< 0.001*	0.96	513.0	< 0.001*	0.98
BDP	3/27	31.6	< 0.001	0.78	18.1	< 0.01	0.67
BAP	5/45	53.8	< 0.001*	0.86	553.2	< 0.001*	0.98
BS × BDP	9/81	5.3	< 0.001	0.37	2.0	0.049	0.18
BS × BAP	15/135	10.1	< 0.001	0.53	95.6	< 0.001	0.91
BDP × BAP	15/135	70.3	< 0.001	0.89	40.4	< 0.001	0.82
BS × BDP × BAP	45/405	8.9	< 0.001	0.50	3.9	< 0.001	0.31

Analyses included repeated-measures factors Ball Speed (BS), Ball Departure Position (BDP) and Ball Arrival Position (BAP). *p*-values with Greenhouse-Geiser corrections are marked with an asterisk (modified degrees of freedom are not reported).

First, orthogonal ball speed affected both the moment of initiation (MoI) and the magnitude of peak velocity (PV) of the interception movements. On average, movement was initiated at 0.42, 0.37, 0.34, and 0.29 s after the onset of ball motion for ball speeds BS1, BS2, BS3, and BS4 (i.e., BFT’s of 2.0, 1.6, 1.2, and 0.8 s), respectively. While movement was thus initiated somewhat earlier for higher ball speeds, the magnitude of PV nevertheless increased with BS: on average, absolute peak velocity was 15.9, 18.0, 22.1, and 30.0 cm/s for ball speeds BS1, BS2, BS3, and BS4, respectively.

**Figure 4:**
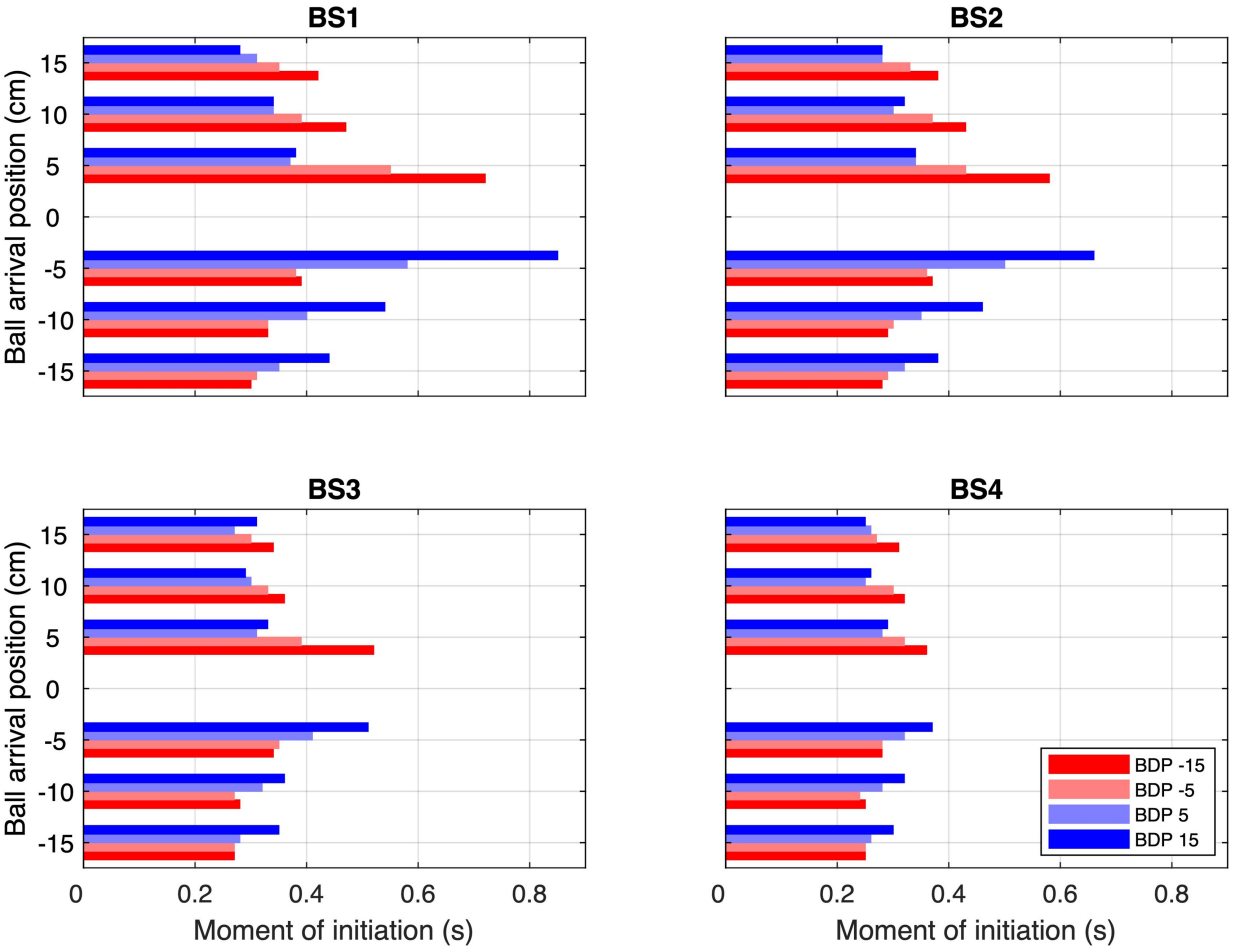
Average moments of initiation (MoI) for each combination of Ball Arrival Position (BAP) and Ball Departure Position (BDP, same color codes as in Figure 3) for each of the four Ball Speed conditions (with ball flight times of 2.0, 1.6, 1.2, and 0.8 s for BS1–BS4, respectively).

**Figure 5:**
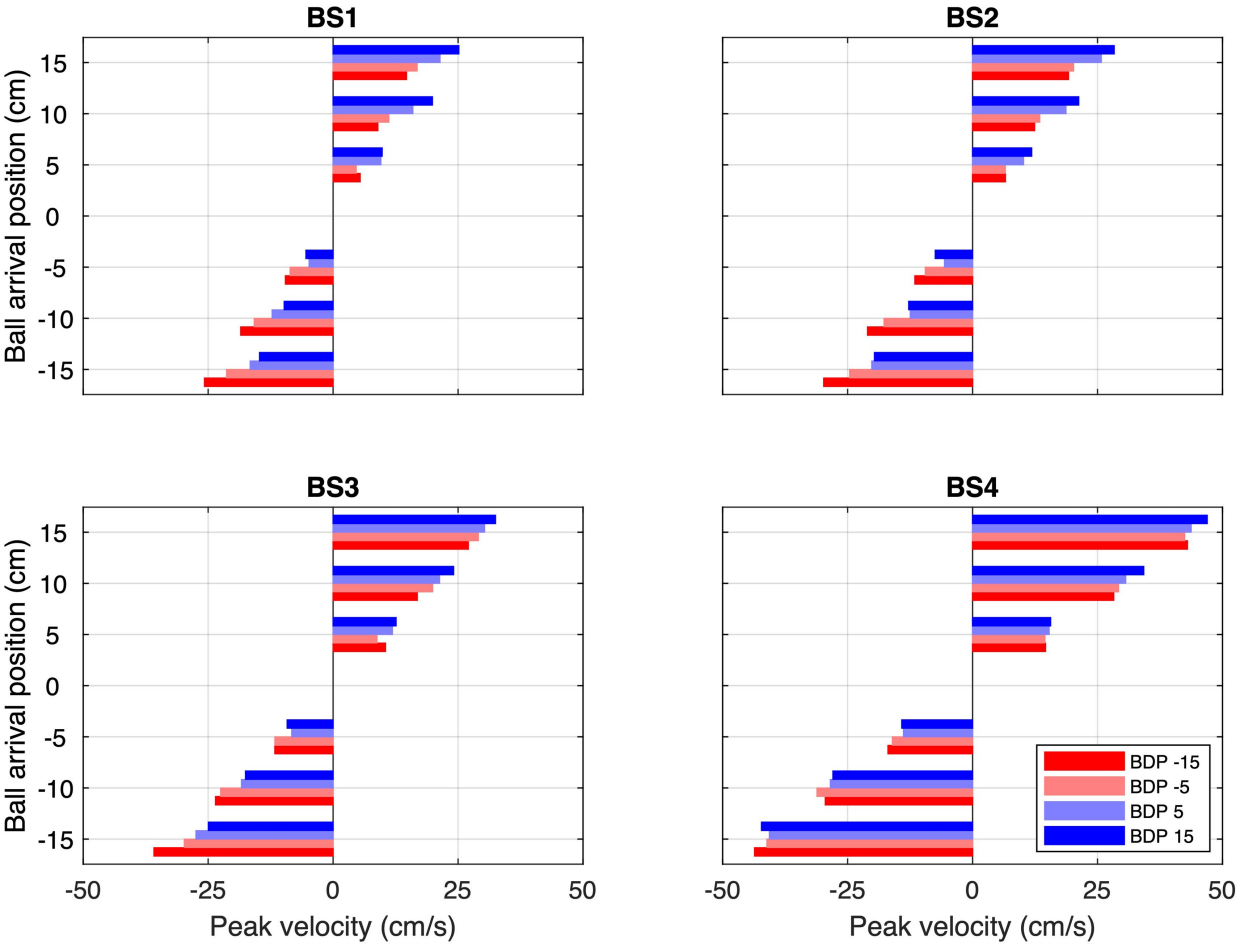
Average peak velocities (PV) for each combination of Ball Arrival Position (BAP) and Ball Departure Position (BDP, same color codes as in Figure 3) for each of the four Ball Speed conditions (with ball flight times of 2.0, 1.6, 1.2, and 0.8 s for BS1–BS4, respectively).

Second, ball trajectory effects were observed at each ball speed. That is to say, we found systematic angle-of-approach effects: ball trajectories converging via different angles of approach onto the same arrival position –different BDPs for same BAPs– gave rise to different stylus movement characteristics, as can already be clearly seen in Figure 3. For trajectories crossing the center of the screen, movements were initiated later than for trajectories remaining on the same side of the screen, as can be confirmed by visual inspection of the location patterns of the dots on individual ball trajectories in the upper parts of each panel in Figure 3, indicating trajectory-average moments of initiation of interceptive movement. This effect was stronger for ball arrival positions closer to the initial stylus position. Indeed, latest initiation was systematically observed for the trajectory conditions BDP = −15 cm to BAP = +5 cm and BDP = +15 cm to BAP = −5 cm, while earliest initiation was systematically observed for trajectory conditions BDP = −15 cm to BAP = −15 cm and BDP = +15 cm to BAP = +15 cm (see Figure 4; for a more detailed boxplot figure, see Supplementary Datasheet 1). A similar pattern of results was observed for peak velocity, with, for identical BAPs, balls crossing the center of the screen giving rise to smaller PV magnitudes than ball remaining on the same side of the screen (see Figure 5; for a more detailed boxplot figure, see Supplementary Datasheet 1). These angle-of-approach effects were most pronounced for the lower ball speeds.

Finally, it is noteworthy that participants sometimes arrived early at the future ball arrival position and then simply remained there to intercept the ball. This behavior of arriving ahead of time was typically observed under the slower ball speed conditions for ball trajectories that did not cross the center of the screen (see Figure 3).

### 3.3 Movement kinematics part 2: balls arriving at the initial stylus position (BAP = 0 cm)

**Figure 6:**
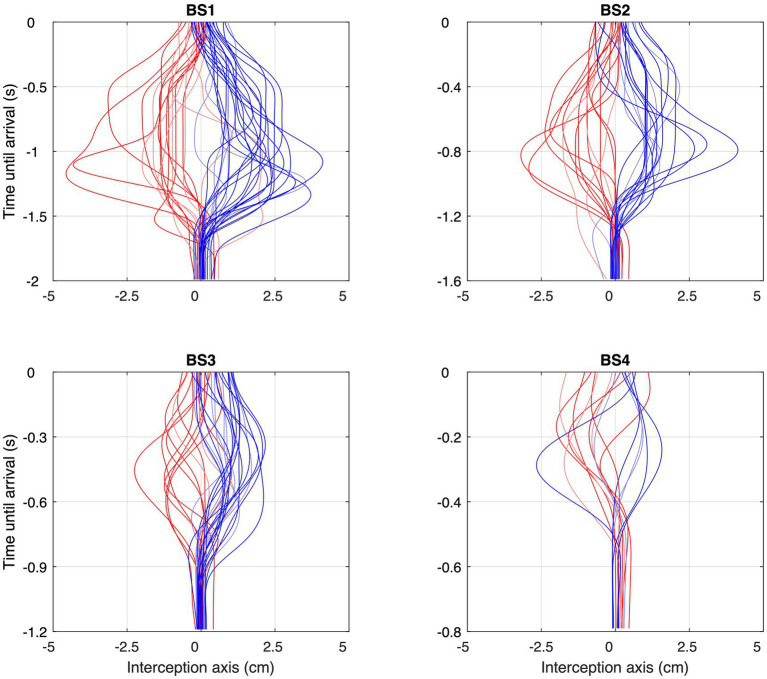
Space–time profiles of the reversal movements observed under each of the four Ball Speed conditions (with ball flight times of 2.0, 1.6, 1.2, and 0.8 s for BS1–BS4, respectively), with red profiles corresponding to balls coming from the left and blue profiles corresponding to balls coming from the right. Trials without reversal movements are not included. Please note the differences in (vertical) time scale in the four panels.

In 480 of the total of 3,360 experimental trials (i.e., 1/7th or 14% of the trials), the ball followed a rectilinear trajectory that arrived at the initial stylus position at the center of the screen. Due to the exclusion of trials with recording problems, 474 trials with BAP = 0 remained available for analysis. We emphasize that in all these BAP = 0 trials, no stylus movement was needed for a successful interception. Yet, reversal movements were observed in 124 (i.e., 26.2%) of these trials. In the grand majority (95.2%) of cases the stylus moved away from the starting position in the direction of the ball departure position (see Figure 6). In all these trials, stylus movement direction subsequently reversed direction, moving back toward the interception location. Reversal movements were observed for all participants, with a between-participant range from 3 to 21 for a median number of 13. As can be seen from Table 2, the number of reversals observed varied with ball speed [*χ*^2^(3) = 15.64, *p* = 0.001] and ball departure position [*χ*^2^(1) = 27.88, *p* < 0.001]. When the ball started from the furthest ball departure positions (i.e., BDP = −15 or + 15 cm) and moved at lowest speed, reversal movements were observed in 50% of the trials, while reversal movement were observed in only 10% of the trials when the ball started from the nearer ball departure positions (i.e., BDP = −5 or + 5 cm) and moved at highest speed.

**Table 2:** Percentage of trials with Ball Arrival Position (BAP) = 0 cm demonstrating reversal movements for each Ball Departure Position (BDP) under each of the four Ball Speed (BS) conditions.

		BDP			
	±15 cm	–5 cm	+5 cm	+15 cm	Mean
BS1	36.7%	24.1%	16.7%	63.3%	**35.3%**
BS2	36.7%	10.0%	20.0%	41.4%	**26.9%**
BS3	34.5%	6.9%	26.7%	48.3%	**29.1%**
BS4	20.7%	10.0%	10.0%	13.3%	**13.4%**
**Mean**	**32.2%**	**12.7%**	**18.3%**	**41.5%**	**26.2%**
		BDP			
		±15 cm	±5 cm		**Mean**
BS1		50.0%	20.3%		**35.3%**
BS2		39.0%	15.0%		**26.9%**
BS3		41.4%	16.9%		**29.1%**
BS4		16.9%	10.0%		**13.4%**
**Mean**		**36.9%**	**15.5%**		**26.2%**

The lower rows present the left/right collapsed data. Means over ball speed conditions are marked in bold.

**Figure 7:**
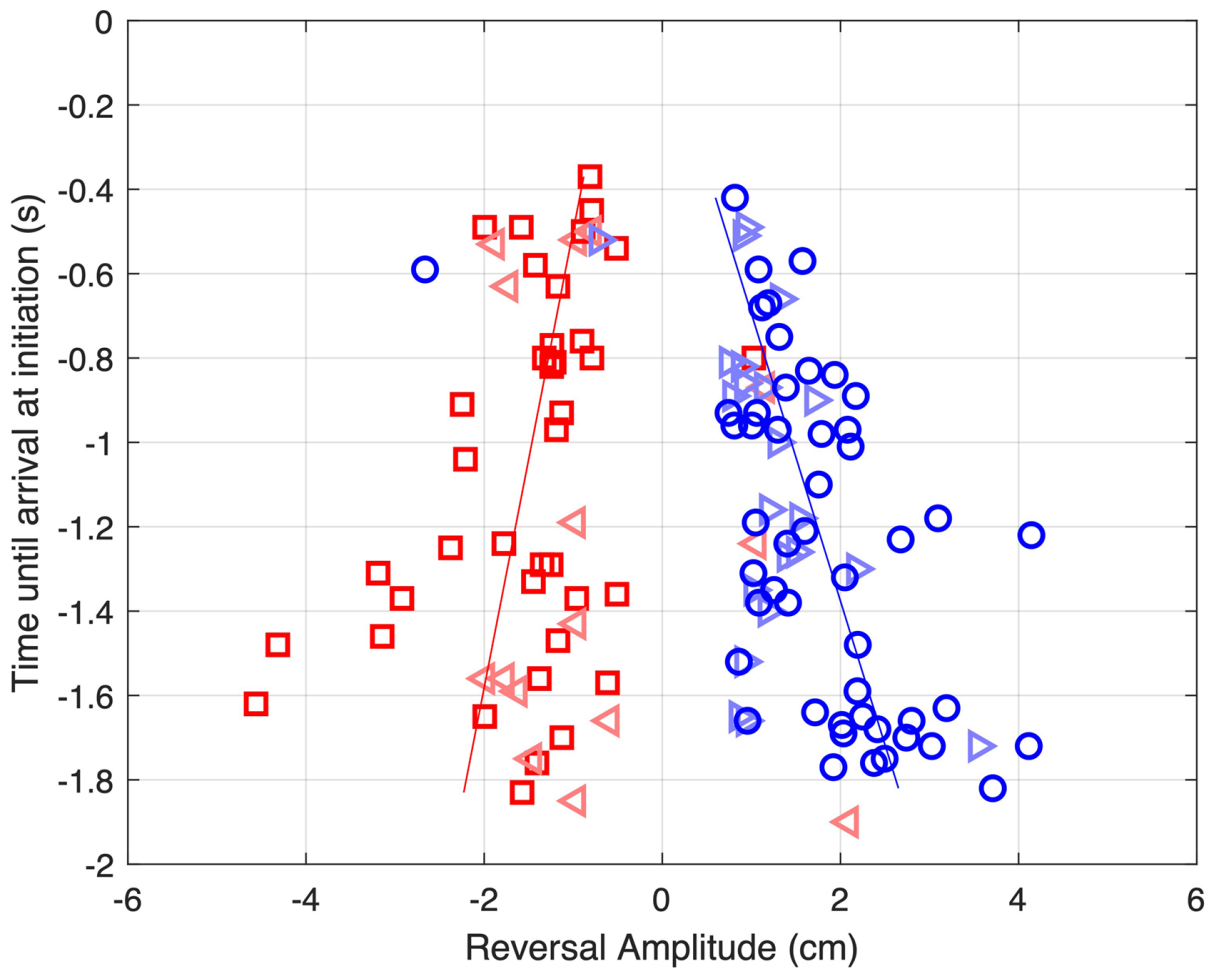
Amplitude of reversal movements as function of time until ball arrival at movement initiation for each of the 124 observed reversal movements for balls arriving at BAP = 0 cm. Red squares: BDP = −15 cm, red triangles: BDP = −5 cm, blue triangles: BDP = +5 cm, blue circles BDP = +15 cm. The lines represent the results of linear regressions for BDP = −15 cm (red) and BDP = +15 cm (blue).

As can be seen from Figure 6, movement reversals did not only show variable amplitudes but also occurred at variable moments before ball arrival. Figure 7 presents all 124 observed movement reversals with their amplitude as a function of time remaining until ball arrival. Interestingly, the amplitude of the reversal movements varied with the timing of movement initiation which was most clearly visible for the larger approach angles. For BDPs −15 cm and + 15 cm, linear regression of amplitude onto time remaining until ball arrival revealed slopes of +0.92 cm/s [*F*(1, 36) = 6.10, *p* = 0.018] and − 1.47 cm/s [*F*(1, 47) = 16.41, *p* < 0.001], respectively. These results indicate that earlier initiation of a reversal movement was accompanied by a larger amplitude.

## 4 Discussion

Studying lateral manual interception for balls approaching in the transverse plane at constant speed along rectilinear trajectories, Montagne et al. (1999) reported two major results. The first, referred to as the *angle-of-approach effect*, was the finding that uniformly moving balls, starting from different departure positions but converging onto the same arrival location on the interception axis and arriving there after the same duration of motion, gave rise to interception movement patterns that systematically varied with the ball’s angle of approach to the interception axis (i.e., the trajectory incidence angle). The second, referred to as the *reversal-movement effect*, was the finding that reversal movements were observed for a substantial proportion of the subset of trials in which the ball moved directly toward the initial hand position. Such reversal movements were typically characterized by a first movement of the hand away from its initial position in the direction of the ball’s departure position, followed by a reversal of the direction of hand movement leading it to return toward its initial position so as attempt to intercept the ball there. The angle-of-approach effect was replicated in subsequent studies of lateral manual interception using rectilinear ball trajectories, both for balls moving in the transverse plane (Arzamarski et al., 2007) and for balls moving in the fronto-parallel plane (Ledouit et al., 2013). Neither of these studies, however, replicated the number or pattern of reversal movements reported by Montagne et al. (1999).

It was thus not surprising that the present study revealed the angle-of-approach effect once again. The effect was indeed directly visible in the ensemble average hand movement trajectories (Figure 3) demonstrating, for each Ball Arrival Position (BAP), a systematic influence of Ball Departure Position (BDP) on their shape. This result was moreover statistically corroborated by the finding of (very) strong effect sizes (η^2^*_p_*s of 0.89 and 0.82, respectively) of the BDP × BAP interaction for the moment of initiation of the interception movement (Figure 4) and the peak-velocity reached during movement (Figure 5). There are, however, two (reviewer-inspired) caveats, both related to the experimental design, that we need to address before drawing definite conclusions. The first is related to the number of participants. It may indeed rightfully be argued that, with only 10 participants, the experimental design of the present study was underpowered (*cf.* Brysbaert, 2019). Notwithstanding such lower-than-normally-desired power, we note that the signature BDP × BAP interactions (capturing the angle-of-approach effect) were nevertheless prominently present. This was also the case in (i) Ledouit et al. (2013), in both their comparable Experiments 1 (*n* = 5) and 4 (*n* = 8) and (ii) Arzamarski et al. (2007) in their comparable Experiment 2 (*n* = 5). With the effect thus being systematically observed, even when statistical power (that is, the likelihood of finding an existing effect) is low, we may conclude that the effect is in fact remarkably robust. The second caveat is related to the present choice (building on Ledouit et al., 2013) of having all ball trajectories start from the same orthogonal (i.e., Y) distance from the interception axis (see Figure 2). In so doing, we were able to control ball flight time (from any given BDP to any given BAP) via (variations in) orthogonal ball speed BS. However, as a result of this choice, within a given BS condition ball speed along the trajectory differed over trajectory incidence angles, thereby constituting a potentially confounding factor in our set-up. Interestingly, such confounding was not present in the experimental set-ups of both Montagne et al. (1999) and Arzamarski et al. (2007), where ball speed along the trajectory was identical over all trajectory incidence angle conditions. Given that both these studies also reported prominent angle-of-approach effects, we may conclude that in and of itself the effect is independent of the absolute magnitudes of ball speed.

The thus evidently robust angle-of-approach effect in the interception of uniformly moving targets implies that interception movements are neither based on *a priori* predictions of when the ball will be where, nor prospectively controlled by information specifying future ball arrival position *XB*_1_ (such as (*dθ/dt)/(dφ/dt*) in the case of ball motion in the transverse plane). Given that Ledouit et al. (2013) demonstrated that the angle-of-approach effect does not result from a perceptual bias in perceived *XB*_1,_ as earlier suggested by Arzamarski et al. (2007), this effect unequivocally demonstrates an influence of information with respect to current lateral ball position (i.e., *XB*_0_), as this is what differentiates the trajectories converging onto the same arrival location on the interception axis.

In order to enhance the chances of replicating the reversal-movement effect, in the present study we sought to reproduce the uncertainty with respect to where the ball might arrive associated with Montagne et al. (1999)’s experimental procedure. To this end, we used a large number of ball arrival positions separated by small distances between adjacent positions. This uncertainty was further enhanced by the particular procedure deployed to bring the hand to its initial position before the start of each trial. As in Montagne et al. (1999), experimental conditions were presented in a randomized order. Finally, we included trials with high ball speeds (i.e., short ball flight times), so as to evoke relatively early initiation of the interceptive action under all ball speed conditions (range effect, Poulton, 1975).

In so doing, the present study revealed that, in the particular situation that the ball will in fact arrive at the initial hand position (BAP = 0 cm), participants did not consistently intercept the ball by simply remaining where they were: in 26% of all these trials, they actually moved away from their initial position before moving back. Under low ball speed and large target incidence angle conditions, this proportion reached 50%. In almost all (95.2%) of the cases such reversal movements were moreover specific to the characteristics of the ball trajectory: balls coming from the left of the initial hand position gave rise to left–right reversal movements, while balls coming from the right of the initial hand position gave rise to right–left reversal movements. Contrary to Arzamarski et al. (2007)’s assertion, the present study therefore confirms that, under certain conditions, direction-specific reversal movements may indeed be evoked in lateral manual interception tasks, as had been reported earlier by Montagne et al. (1999). We note that, as we did not deploy a dedicated evaluation protocol, we cannot assert which of the procedural changes with respect to the Ledouit et al. (2013) and Arzamarski et al. (2007) studies (i.e., guiding the hand to the starting position, including more potential ball arrival positions and including higher balls speeds), alone or in combination, was particularly influential in eliciting the coming to the fore of a consequential number of reversal movements in the present study. All we may conclude is that together they did lead to that result.

As for the angle-of-approach effect discussed earlier, the reversal-movement effect observed in the present study and by Montagne et al. (1999) again implies that interception movements are not based on *a priori* predictions of when the ball will be where, nor on a prospective control strategy uniquely relying on information specifying future ball arrival position *XB*_1_. Given the demonstrated influence of ball position, both sets of results thus imply that information with respect to current lateral ball position *XB*_0_ plays a role. Indeed, with balls coming from the left of the initial hand position typically giving rise to left–right reversal movements and balls coming from the right of the initial hand position typically giving rise to right–left reversal movements, the observed reversal movements were quite consistently specific to the direction from which the ball came. Moreover, with reversal movements typically revealing larger amplitudes for ball trajectories starting from (and therefore during approach remaining at) a larger distance from the initial hand position, the observed reversal movements amplitudes were indeed quite consistently specific to the lateral distance of the ball.

**Figure 8:**
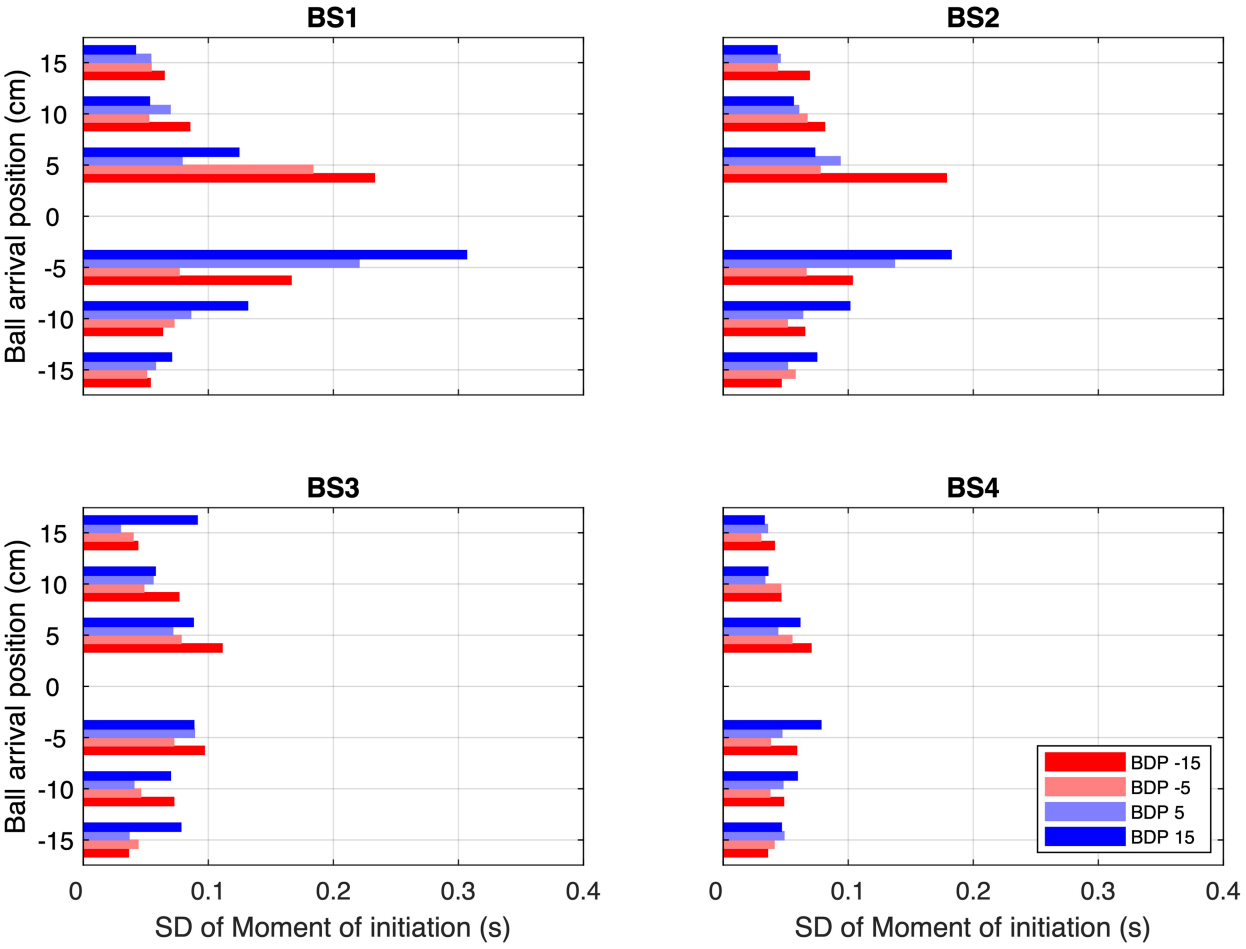
Standard deviations over all trials of all participants of the moments of initiation (MoI) of interceptive movement for each combination of Ball Arrival Position (BAP) and Ball Departure Position (BDP, same color codes as in Figure 3) for each of the four Ball Speed conditions (with ball flight times of 2.0, 1.6, 1.2, and 0.8 s for BS1–BS4, respectively).

However, prospective control relying on uniquely *XB*_0_-specific information cannot explain the full set of results either, since reversal movements occurred regularly but not systematically on all trials as would then be expected. How then might we understand the observed frequencies of reversal movements? We suggest a two-step explanation. The first step is to not limit the information used to either being specific to current ball position (zeroth-order *XB*_0_) or to future ball position (first-order *XB*_1_), but to allow intermediate states (orders). Such intermediate states can be either conceived as resulting from a combination of informational quantities of different temporal orders (e.g., Craig et al., 2009; Dessing et al., 2002, 2005) or as an informational quantity of a fractional order (Jacobs et al., 2012; Bootsma et al., 2016). The computationally simplest way of combining (time-varying) *XB*_0_ and (constant) *XB*_1_ would be to consider *XB_α_*(*t*) = *XB*(*t*) + α · *dXB*/*dt* · *t*, where α = 0 yields *XB*_0_ and α = 1 yields *XB*_1_. Intermediate states would then be characterized by 0 < α < 1. The second step is to allow for noise-induced fluctuations in the operative threshold for movement initiation. Although we take such a threshold to be related to the required action (see Bootsma et al., 2016) rather than being purely informational, under each experimental condition it would still be co-determined by such a *XB_α_*-specific information source. As a result of the presence of noise-induced fluctuations in the operative threshold, the moment at which movement is initiated would then vary to a certain degree over (and even within) trials within the same condition. In order to pinpoint such fluctuations, we calculated the observed variability in the moment of movement initiation for all conditions requiring movement in order to intercept the ball (i.e., for all BAPs except BAP = 0 cm). Interestingly, inspection of these variabilities under the different experimental conditions (see Figure 8) revealed that variability was highest (and of considerable magnitude, especially for the lower ball speeds) for ball trajectories crossing the center of the screen while arriving close to the initial hand position (i.e., from BDP = −15 or − 5 cm to BAP = +5 cm and from BDP = +5 or + 15 cm to BAP = −5 cm.). Thus, by inference, balls moving toward BAP = 0 cm (i.e., the initial hand position) may also be expected to have given rise to considerable fluctuations, both over and within trials, in the operative threshold for movement initiation. With balls coming from BDPs ±15 and ± 5 cm, in this BAP = 0 cm condition the magnitude of *XB*_α_ declines over time but does not become zero until the ball reaches the interception axis. In the presence of considerable fluctuations in the threshold for the BAP = 0 cm conditions, in these conditions the fluctuating *XB*_α_-dependent threshold may then be expected to be reached on certain trials and not on others, resulting in movements initiations on some trials and not on others.

Due to the magnitude of *XB*_α_ declining more slowly over time for the lower ball speeds, the percentage of trials on which the fluctuating threshold would be reached would then be larger than for the higher ball speeds, corresponding to the pattern of results observed (see ball speed effect in Table 2). In the same line of reasoning, due to the larger absolute magnitudes of *XB*_α_ for balls starting from further distances to the future ball arrival position (i.e., conditions with BDP = −15 and + 15 cm), the percentage of trials on which the fluctuating threshold would be reached would be larger for these conditions than for those with balls starting from the closer distances (i.e., conditions with BDP = −5 and + 5 cm), once again in line with the pattern of results observed (see BDP effect in Table 2). Finally, as the magnitude of *XB*_α_ declines over time for all BAP = 0 cm trajectory conditions, this line of reasoning is also compatible with the finding that earlier initiated reversal movements were associated with larger reversal amplitudes (see Figure 7).

It is important to realize that reliance on information specific to some partial combination of ball position and velocity (such as *XB*_α_ where 0 < α < 1) also allows the angle-of-approach effect to emerge, as it retains a particular role for the ball’s current lateral distance (and is therefore *XB*_0_-related).

We note that reversals in movement direction have recently also been evidenced in the interception of uniformly moving targets in the framework of locomotor interception by steering (Ceyte et al., 2021; Casanova et al., 2022). These studies revealed reversals in the direction of steering under particular combinations of lateral target departure position and inward target speed. In the framework of the current contribution, it is remarkable that striking similarities with our present findings were reported in these studies: (i) reversals were found regularly, but not systematically on all trials of the relevant conditions, (ii) reversals were consistently specific to the direction (left or right with respect to the participant’s initial heading direction) from which the target came, (iii) more reversals were found for targets starting from larger sideward distances, and (iv) reversals of larger amplitude were found for earlier initiated steering actions. In locomotor interception, implying movement of the point of observation, the optical information used to guide action appears to reside in (changes in) the target’s azimuthal bearing angle (Fajen and Warren, 2007; Bootsma et al., 2016; Ceyte et al., 2021; Casanova et al., 2022). Not surprisingly therefore, Ceyte et al. (2021) concluded that participants relied on some kind of combination of the target’s bearing angle-related (zeroth-order) and angular rate of change-related (first-order) information, which, they suggested, could take the integrative form of a fractional-order, slightly below 1, as suggested by Bootsma et al. (2016).

Locomotor interception, however, differs from manual interception in an important way, as –in their pure forms– the former involves controlling movement of the point of observation, while the latter involves controlling movement of the hand. In locomotor interception whole-body movement directly affects the target’s bearing angle. Since this angle is defined with respect to the point of observation, hand movement does not have this same effect and a different source of information is therefore required in manual interception. As already alluded to in the Introduction section, in the case of ball motion in the transverse plane, information with respect to ball position, whether it be with respect to *XB*_0_, *XB*_1_ or *XB*_α_, is available at the point of observation via the ratio of different rate-of-change orders of the ball’s optical bearing angle *θ* to the ball’s optical size *φ* [i.e., zeroth-order *θ/φ =* (*d^0^θ/dt^0^)/(d^0^φ/dt^0^*), first-order (*d^1^θ/dt^1^)/(d^1^φ/dt^1^*) and αth-order (*d^α^θ/dt^α^)/(d^α^φ/dt^α^*)] (see Podlubny, 1999, 2002, for information on fractional derivatives). In the case of ball motion in the fronto-parallel plane, as in the present study, no informational equivalent has so far been identified. Benerink et al. (2016) suggested an alternative starting point for the information used in such a setting, based on the rate of change in the optical angle *β* formed by the interception axis and the line connecting the ball and the end-effector (here stylus position): as future interception would be guaranteed by a constant *β*-angle, control could be accomplished by continuously seeking to null *dβ/dt* (or *dβ^α^/dt^α^*, for that matter). However attractive such a *β*-based prospective control strategy might appear to be at first sight, it cannot account for the finding that, for low ball speeds, the end-effector regularly reaches (and subsequently remains at) the future interception point before the ball arrives there (see Figure 3 for such early arrival behavior). We therefore suggest that a perspective incorporating informational constraints into an appropriate movement dynamics (e.g., Schöner, 1994; Schöner and Santos, 2001; Borooghani et al., 2024) is to be pursued in future work.

For the time being we conclude that interception of uniformly-moving targets, whether it be in lateral manual interception tasks or in a locomotor interception-by-steering tasks, is prospectively guided by information of an intermediate order (between 0 and 1), as only such a control scheme allows capturing the distinctive qualitative aspects of the interception movements observed here and in the literature (Montagne et al., 1999; Arzamarski et al., 2007; Ledouit et al., 2013; Bootsma et al., 2016; Ceyte et al., 2021; Casanova et al., 2022). In the present framework, guidance by such information of an intermediate order indeed allows an explanation for the emergence of both the angle-of-approach effect and the reversal-movement effect. It even allows understanding of the fact that the reversal movements do not occur systematically on all trials of a given condition, but do occur more often (and are of larger magnitude) under certain ball-trajectory conditions (large incidence angles and slow ball speeds) than others (small incidence angles and high ball speeds). Whether manual interception of non-uniformly moving (e.g., curving) ball trajectories would be guided by information of a higher intermediate order (between 1 and 2), as has been reported for locomotor interception (Bootsma et al., 2016; van Opstal et al., 2022; van Opstal et al., 2023), remains an open question at present.

## Data Availability

The raw data supporting the conclusions of this article will be made available by the authors, without undue reservation.
