# Supplementary material for: Angle-of-approach and reversal-movement effects in lateral manual interception
Source: Front Psychol. 2024 Nov 7;15:1433803. doi: 10.3389/fpsyg.2024.1433803 (PMC11580702; doi:10.3389/fpsyg.2024.1433803)

Supplementary Material

Angle-of-approach and reversal-movement effects in lateral manual interception

Simon Ledouit, Danial Borooghani, Remy Casanova, Nicolas Benguigui, Frank T.J.M. Zaal, and Reinoud J. Bootsma

Supplementary Figure 1. Boxplots of average moments of initiation (MoI) for each combination of Ball Arrival Position (BAP) and Ball Departure Position (BDP, same color codes as in Fig. 3) for each of the four Ball Speed conditions (with ball flight times of 2.0, 1.6, 1.2 and 0.8 s for BS1 to BS4, respectively).

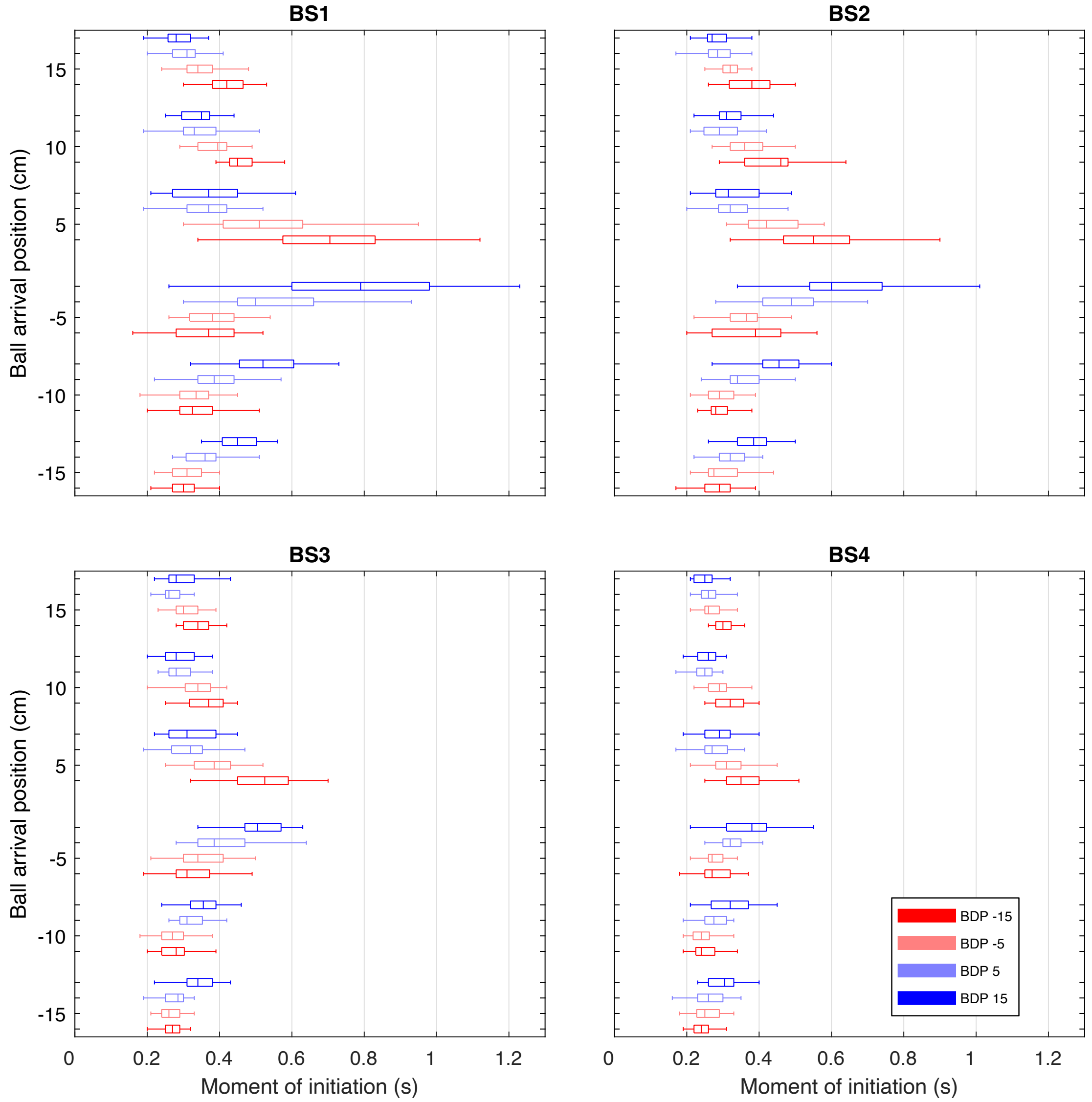

Supplementary Material

Angle-of-approach and reversal-movement effects in lateral manual interception

Simon Ledouit, Danial Borooghani, Remy Casanova, Nicolas Benguigui, Frank T.J.M. Zaal, and Reinoud J. Bootsma

Supplementary Figure 2. Boxplots of average peak velocities (PV) for each combination of Ball Arrival Position (BAP) and Ball Departure Position (BDP, same color codes as in Fig. 3) for each of the four Ball Speed conditions (with ball flight times of 2.0, 1.6, 1.2 and 0.8 s for BS1 to BS4, respectively).

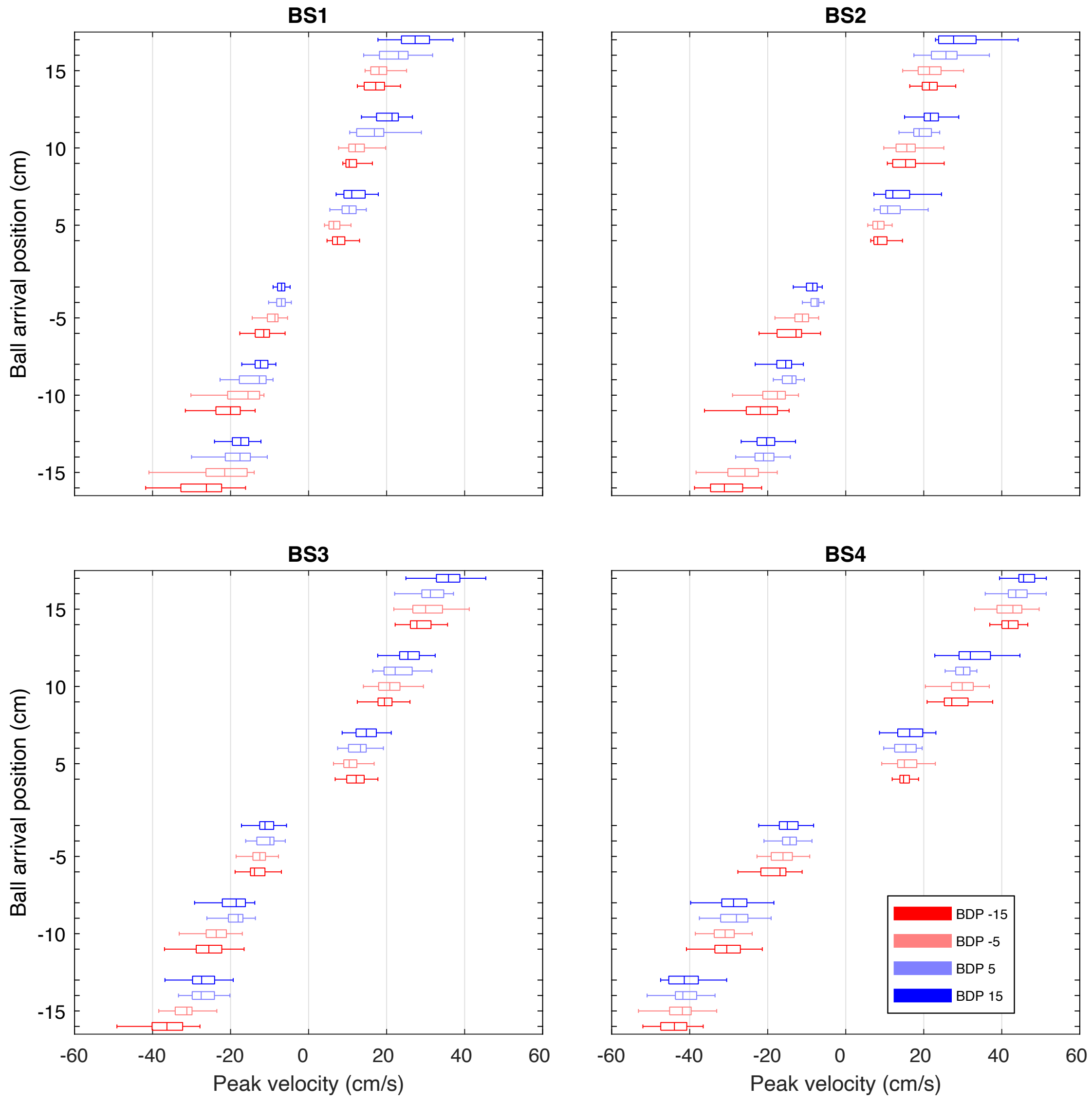

Supplement: Supplementary file 1 [file Data_Sheet_1.PDF]
